# Variations induced by body weight and background lesion normalization in standardized uptake value estimated by F18-FDG PET/CT

**DOI:** 10.1186/s41824-022-00142-5

**Published:** 2022-10-13

**Authors:** Eman M. Badawe, Hesham Abdel Gawad, Mohamed S. El-Nagdy, Magdy M. Khalil

**Affiliations:** 1 Department of Physics, Faculty of Science, Helwan University, Cairo, Egypt; 2 Faculty of Medicine, Kasr Al-Aini Center for Nuclear Medicine and Oncology (NEMROK), Cairo University Hospitals, Cairo University, Giza, Egypt; 3 School of Biotechnology, Badr University in Cairo (BUC), Badr City, Cairo Egypt

**Keywords:** PET/CT, SUV normalization, Body weight, Lean body mass, Body surface area, Body mass index

## Abstract

**Aim:**

This work aims to study the impact of different SUV variants in terms of mean and maximum measures as well as various normalization methods with respect to body weight, body mass index, body surface area, and lean body mass in patients with lymphoma.

**Methods:**

Sixty-nine patients (34 male–35 female) were retrospectively selected. All patients had undergone F18-FDG PET/CT using the standard imaging protocol. In the first part of this study, SUVmean and SUVmax of patients’ lesions and three background sites including liver, aorta, and muscle were determined. Then, the normalization of lesion SUV to body weight and body background sites was performed. The ratio of lesion SUVmax to body background sites (muscle, aorta, and liver) SUVmax was determined in addition to the ratio of lesion SUVmean to body background sites SUVmean. The second part of the study included the calculations of the body mass index (BMI), body surface area (BSA), and lean body mass (LBM). The normalization of lesion, liver, aorta, and muscle SUV to BMI, BSA, and LBM was calculated and compared to each other.

**Results:**

After performing the appropriate statistical calculations, the results showed that there is a significant difference in SUV measurements between the three background sites. Lesions normalized to the liver were significantly lower than those normalized to aorta and muscle and the results also showed a higher magnitude of lesions normalized to muscle in comparison to the aorta. The SUVmax and SUVmean normalized to different body weight indices showed the lowest variation with BSA and BMI while being increasingly higher with lean body mass using the two methods James and Janmahasatian, respectively, and then highest with body weight.

**Conclusion:**

The SUVmax and SUVmean showed lower variance in comparison to other background regions. Less variation was also remarkable in SUVmean normalized to BSA and Janma lean mass and also when SUVmax is normalized to James lean body mass. The SUVmax normalized to lean (i.e., James) as well as SUVmean normalized to lean (i.e., Janma) and BSA showed a significant independence with body weight.

## Introduction

There are many methods for measuring the metabolic activity of F18-Fluorodeoxyglucose (F18-FDG) accumulation in tumors. PET scanners are designed to measure the in vivo radioactivity concentration [e.g., kBq/ml], which is directly linked to the FDG concentration. This is the relative tissue uptake of FDG that is of clinical interest (Kinahan and Fletcher 2010). A significant advantage of (PET) is the ability to measure radiotracer accumulation.

Standardized uptake value (SUV) is the most widely used parameter to evaluate the accumulation of tracer in PET studies (Eq. 1) (Adams et al. 2010).

SUV is a semiquantitative measure of the FDG uptake, and hence an estimate of glucose metabolic activity,

1 $${\text{SUV}} = \frac{{{\text{Activity}}\; {\text{Concentration}}\;{\text{ in}}\;{\text{ tissue }}\;\left( {{\text{voxel}}\;{\text{ or}}\;{\text{ VOI}}} \right)}}{{{\text{Normalized}}\;{\text{ injected }}\;{\text{Activity}}\; \left( {{\text{bw}},\;{\text{lbm}}\;{\text{ or}}\;{\text{ bsa}}} \right)}}$$

To evaluate SUV, a 2D or 3D region of interest (ROI) is placed centrally within a target (i.e., tumor) using an interactive workstation. The measured radioactivity within the ROI is normalized to the average radioactivity concentration within the body that can be approximated as the injected dose divided by patient body size. SUV can take a variety of forms when normalizing the measured uptake to the patient body including body weight (bw), body surface area (bsa), lean body mass (lbm), and body mass index (bmi). The former has been the most common variant of SUV measurements widely adopted among clinical institutions (Paquet et al. 2004).

The most frequently used SUV metrics are SUVmax and SUVmean. SUVmax measurements take into account the maximal pixel concentration in the chosen lesion. It is sensitive to noise since it increases positive bias as noise increases and a topic of debate in treatment response assessment. The variability of SUVmax which can be related to image noise accounts for half of the actual variability as reported in one study (Basu et al. 2014). SUVmean is more variable because of operator-dependent factors such as shape and size of mask delineation and location within or around a lesion, and also the presence of tumor heterogeneity and variable level of background 18 F-FDG activity (Khalil 2016).

### SUV normalization

SUV can be normalized to body weight (Eq. 2), body surface area according to DuBois Formula (Schmidt 2019) (Eq. 3), body mass index (Kolimechkov and Petrov 2020) (Eq. 4), and lean body mass which calculated from predictive equations through parameters such as sex, height, and the body weight (Halsne et al. 2018). Lean body mass can be estimated by James equations (Eq. 5) which rely on the square of the weight with a negative coefficient or by Janmahasatian equations (Eq. 6) which based on bioelectrical impedance analysis (Tahari et al. 2014).

2 $${\text{SUV}}\;\left( {{\text{bw}}} \right) = \frac{{{\text{radioactive}}\;{\text{ concentration}}\;{\text{ in}}\;{\text{ tissue}}}}{{{\text{injected}}\;{\text{ dose/body}}\;{\text{ weight}}}}$$

3 $${\text{BSA}}\;\left( {{\text{m}}^{2} } \right) = \sqrt {\frac{{\left[ {{\text{height}}\;\left( {{\text{cm}}} \right)\; \times \;{\text{weight}}\;\left( {{\text{kg}}} \right)} \right]}}{3600}}$$

4 $${\text{BMI}}\;\left( {{\text{kg/}} {\text{m}}^{2} } \right) = \frac{{{\text{weight}}\;\left( {{\text{kg}}} \right)}}{{\left( {{\text{height}}\;\left( {\text{m}} \right)} \right)^{2} }}$$

5 $${\text{LBMjames}} = \left\{ {\begin{array}{*{20}l} {1.1\; \times \;{\text{BW}} - 128\; \times \; \left( {\frac{{{\text{Bw}}}}{{{\text{Height}}}}} \right)^{2} {\text{Men}}} \hfill \\ {1.07\; \times \;{\text{BW}} - 148\; \times \;\left( {\frac{{{\text{BW}}}}{{{\text{Height}}}}} \right)^{2} {\text{Women}}} \hfill \\ \end{array} } \right.$$

6 $${\text{LBMjanma }} = \left\{ {\begin{array}{*{20}l} {\frac{{9.27\; \times \;10^{3} \; \times \;{\text{BW}}}}{{6.68\; \times \;10^{3} { } + { }216 \times {\text{BMI}}}} {\text{Men}}} \hfill \\ {\frac{{9.27\; \times \;10^{3} \; \times \;{\text{BW}}}}{{8.78\; \times \;10^{3} { } + { }244\; \times \;{\text{BMI}}}} {\text{Women}}} \hfill \\ \end{array} } \right.$$

The SUV is affected by many technical and physiologic aspects such as uptake time, body composition plus blood glucose level (Boellaard 2009). These factors may cause significant variability in SUV measurements and recent publications were made to standardize quantitative PET imaging that consists of recommendations and guidelines to reduce variability in the SUV and enhance the accuracy of results across different institutions (Shankar et al. 2006; Boellaard et al. 2008). One method of minimizing SUV variability is by correcting lesion uptake for the level of background uptake.

However, this study was undertaken to tackle great part of these variabilities especially those related to individual patients by considering different background sites as well as different body weight regimes as normalizing factors. The former would be able to account for different metabolic variations, different effects of drugs, and probably any medical or pharmaceutical intervention that patients may have had. The problem of reproducibility may not solely be resolved by SUV to background ratios. However, a significant component of reproducibility could be well adjusted and fixed with proper control of other confounders.

Many background sites have been used including liver, mediastinal blood pool, and internal jugular vein (Azmi et al. 2017). The purpose of this study was therefore to investigate the impact of different SUV variants in terms of mean and maximum measures as well as various normalization methods with respect to body weight, body mass index, body surface area, lean body mass in patients with lymphoma.

## Materials and methods

### Patients

The present study has covered patients who were referred to initial diagnosis and suspicious of lymphoma. Patients with lymphoma were selected between 2017 and 2019 from electronic data records. The total number of patients selected was 69 who had undergone F18-FDG PET/CT using the standard imaging protocol. Patient characteristics are summarized in Table 1.

**Table 1:** Patient’s characteristics

Mean	
Gender	34 Male
	35 Female
Weight (Kg)	81.58 ± 21.27
Height (m)	1.62 ± 0.09
Injected dose (mCi)	8.68 ± 2.12
Uptake time (hr)	1.30 ± 0.44
BMI	24.2﻿ ± 1.86
*LBM*	
James	4.07 ± 3.07
Janma	3.99 ± 2.92
BSA	1.5 ± 0.11

*N* = 69 patients (35 females, 50.7% and 34 males, 49.3%)

### Patient preparation

The standard patient preparation adopted in our institution was followed and made uniform across the whole study population. Patients were told to fast for at least 4–6 prior to administration of FDG. They were instructed not to perform any heavy muscular activity 24 h before the exam to avoid extraneous uptake and reduce imaging background. Furthermore, considerable hydration was recommended by intake of 1 L of water within 2 h before administration of FDG.

In all patients, height and weight have been measured and recorded. Blood glucose level was also measured to determine if the concentration within the reference range (< 120 mg/dl) for nondiabetic patients and (150–200 mg/dl) for diabetic persons. If the serum glucose concentration was greater than the normal range, a rescheduling was considered. Full patient history was taken including information of prior treatment with chemotherapy, radiation, or any other experimental therapeutics.

Patients were asked to urinate shortly before entering the PET examination room. Waiting conditions, preparation room, and room temperature were comfortable to permit the best resting conditions during and after FDG administration to reduce muscle and brown-fat uptake.

### Imaging protocol

Patients were scanned in straight supine position with arms up and head first mode starting with CT scanning for attenuation correction and then the bed shuttle was let to move to start the PET portion of the procedure. Patients were instructed not to move during the imaging session with shallow breathing. Metals and other attenuating materials were kept away to avoid attenuation artifacts and maintain image quality. Emission data were acquired within 55–65 min after administration of F18-FDG.

Decay times and correct isotope was entered in the acquisition computer. The dose calibrator was cross calibrated with scanner time to maintain accurate measurements. Other relevant information was fed into the acquisition workstation including patient weight, height, injection time, and injected dose.

The time per bed was set at 3 min/position. The average injected radioactivity was 8.68 mCi of 18F-FDG. The amount of injected radioactivity was routinely recorded using the radioactivity of the syringe measured before and after injection, see Table 1. The amount of injected radioactivity was routinely measured through quantitation of the radioactivity of the syringe before and after injection.

### Image reconstruction

A 576-mm FOV was used in all scans providing a volume dimension of 144 × 144 mm or a voxel size of 4 × 4 × 4 mm^3^. Images were reconstructed using a TOF, list-mode, blob-based, ordered subset maximum-likelihood expectation–maximization algorithm (TOF-OSEM).

The corrections were carried out in the reconstruction model accounted for detector efficiency using a component-based method; scatter using a combination of single scatter and Monte Carlo simulation, and random corrections were carried out using smoothed delay-line coincidence data. The reconstruction software compensates for changes in TOF resolution as a function of measured detector count rate by setting the TOF kernel width based on the average singles rate in each frame.

The TOF resolution was determined based on the average singles rate within that frame. For those 69 patients we will measure both SUVmax and SUVmean for lesion, and muscle, aorta, and liver as a background. Then, these measurements will be normalized to body weight, body mass index, body surface area, and lean body math.

### Image analysis

All the PET/CT studies were retrieved from the electronic archival system and then prospectively examined on the workstation. PET, CT, and also fused PET/CT images were carefully reviewed on the three major planes including axial, coronal, and sagittal planes.

For the purpose of this study, the maximum and mean SUVs were measured for the primary gross tumor volume. Lesion demarcation was based on visually observing the region on the PET images that have the most intense FDG uptake using the CT images as anatomical guide in all patients' diagnosis.

### Lesion segmentation

To obtain the optimum values for SUV measurements, a 3D fitting function was generated so that we can estimate the appropriate threshold value of a given PET lesion when background contrast and lesion CT volume are provided (Abdel Gawad et al. 2019). A logarithmic data fit using the commercially available ThreeDify ® Excel add-ins was performed (https://threedify.com/free-excel-3d-add-trial/). The following equation has resulted from the fitting process:

7 $$z = 72.55 - 1.799\ln \left( x \right) - 11.33\ln \left( y \right)$$

where *x* is the lesion 2D volume, *y* is the background contrast and *z* is the threshold value. As lesion 2D volume is spherical (i.e., $= \frac{4}{3}\pi r^{3}$) and sphere diameter (*D*) = 2*r*, Eq. 8 can be reformulated as follows using diameter instead of volume:

8 $$z = 73.714 - 5.397\ln \left( D \right) - 11.33\ln \left( y \right)$$

where *y* is the background contrast and *D* is the lesion 2D diameter while z is the threshold. After we determine the appropriate threshold value, a 3*D* ROI can be generated on the lesion volume so that the SUV values can be evaluated.

### Statistical analysis

Data were presented as mean ± sd or median based on whether data are normally distributed or not, respectively. In the first part of this study, normalization of lesion SUV to body weight and body background sites, the ratio of lesion SUVmax to body background sites (muscle, aorta, and liver) SUVmax was determined in addition to the ratio of lesion SUVmean to body background sites SUVmean. The second part of the study included the calculations of the body mass index (BMI), body surface area (BSA), and lean body mass (LBM).

The normalization of lesion SUV to BMI, BSA, and LBM was calculated based on the following; the value of lesion SUVmax and lesion SUVmean for a particular patient was divided by patient body weight to exclude it from the formula and then the resulting value was multiplied by the values of BMI, BSA, and LBM based on equations described above. This step was repeated for muscle, aorta, and liver. Microsoft Excel version 2010 and Minitab 19 were used to enter the data into a spreadsheet and perform statistical analyses. Statistical software package for social sciences (SPSS, version 25, Chicago, inic) was used in all statistical analyses considering a *p*-value of less than 0.05 statistically significant.

The nonparametric Friedman test and Kruskal–Wallis (one-way ANOVA on ranks) were used to test the mean ranks among the different groups. The Wilcoxon sign test was then used as post hoc for pairwise comparison of the lesion and normalized data sets. A *p*-value less than 0.05 was considered statistically significant and a multiple comparison significance of 0.017 was applied using Bonferroni correction. Spearman correlation was used to interrogate the relationship between individual index and lesion SUV normalized to different body weight indices.

The Spearman correlation was used due to the non-normality of the dependent variable (i.e., SUV measurements).

## Results

### Comparison of background sites

**Fig. 1:**
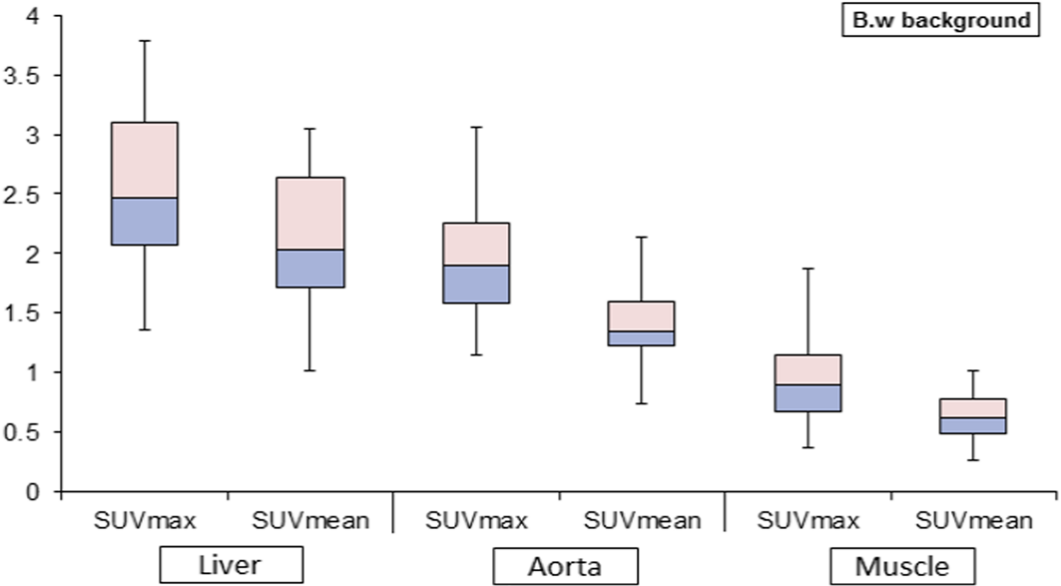
The average SUV value of liver, aorta, and muscle for lymphoma patient group as a background using SUV max and SUVmean

The three background sites showed a statistically significant difference in SUVmax and SUVmean measurements (both *p* < 0.001). Table 2 describes mean and max SUVs of tumor lesions, liver, aorta, and muscle tissues measured for the patient population, whereas Fig. 1 demonstrates the magnitude of variation and the relative relationship among the three different background regions. Pairwise comparison using Wilcoxon sign test demonstrated a significant difference between each pair of the background sites. The liver background in terms of SUVmax and SUVmean was significantly higher than that values produced by aorta and muscle background (*Z* = − 5.6 and − 9.9, *p* < 0.001) and (*Z* = − 6.9 and − 10.0, *p* < 0.001). The aorta was also significantly higher than muscle in the two SUV metrics (*Z* = − 8.7 and − 7.2, respectively, both *p* < 0.001).

**Table 2:** Statistical descriptions of patients SUVmax, SUVmean including tumor lesions, liver, aorta, and muscle tissues

	Lesion	Liver	Aorta	Muscle
*SUVmean*				
Mean	6.29 ± 4.66	2.10 ± 0.52	1.40 ± 0.35	0.64 ± 0.23
Median	5.69	2.05	1.35	0.61
Max	20.48	3.05	2.15	1.78
Min	0.18	1.02	0.74	0.26
*SUVmax*				
Mean	12.58 ± 11.43	2.51 ± 0.59	1.93 ± 0.43	0.93 ± 0.36
Median	9.91	2.49	1.85	0.85
Max	50.8	3.78	3.06	2.26
Min	0.27	1.36	1.15	0.37

### Lesion normalized to different background

Table 3 describes lesion normalized to liver, aorta, and muscle background regions. After SUVmax lesion normalization with respect to the three background sites, the Friedman test showed a significant (*χ*^2^ = 127.0, *p* < 0.001) difference in the mean ranks of the three different types of lesion normalization. The calculated medians of SUVmax (i.e., Interquartile with 25th and 75th quartiles) of the normalized lesion were 3.5 (1.4, 8.5), 5.1 (1.8, 9.6), and 10.4 (4.3, 23.2) for liver, aorta, and muscle, respectively. Lesions normalized to the liver were significantly lower than those normalized to aorta and muscle (*Z* = − 5.8 and − 7.2, *p* < 0.001). The pairwise test also showed a higher magnitude of lesions normalized to muscle in comparison to aorta (*Z* = − 7.2, *p* < 0.001).

**Table 3:** Statistical descriptions of normalization of lesion with respect to different backgrounds

	Lesion/Liver	Lesion/Aorta	Lesion/Muscle
*SUVmean*			
Mean	3.38 ± 2.88	4.86 ± 4.06	10.90 ± 9.35
Median	2.76	4.04	8.66
Max	13.0	20.7	44.9
Min	0.09	0.14	0.31
*SUVmax*			
Mean	5.61 ± 5.80	6.84 ± 6.39	15.0 ± 13.9
Median	3.52	5.06	10.43
Max	31.9	27.6	57.5
Min	0.10	0.18	0.30

**Fig. 2:**
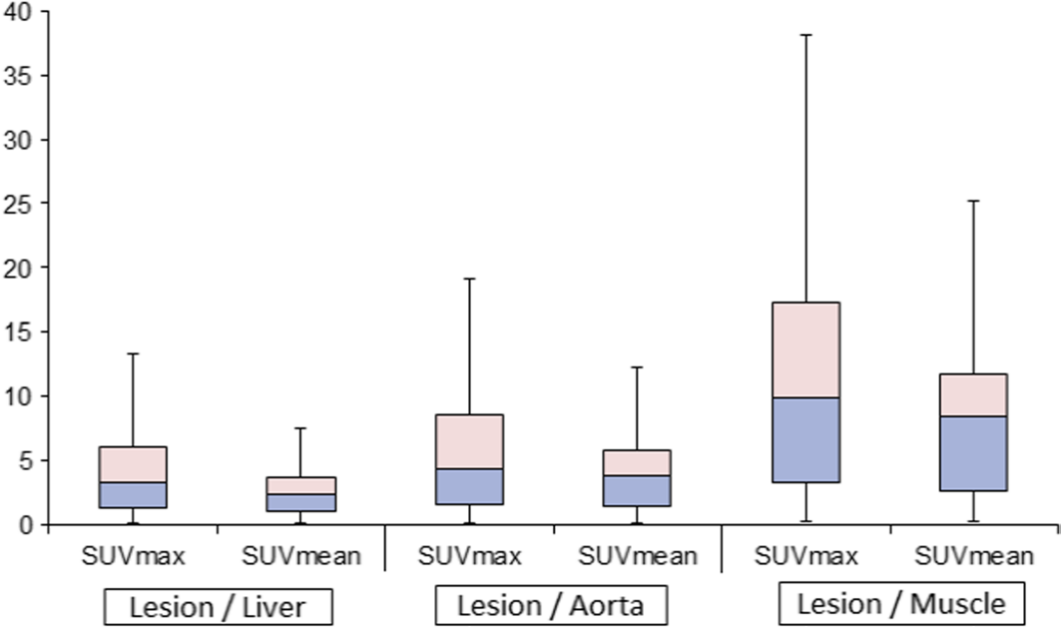
The lesion SUV normalization to different background areas including liver, aorta, and muscle using SUVmax and SUVmean

The same was true for SUVmean measurements, there was a significant difference among the three types of normalization (*p* < 0.001). Pairwise comparison showed an increased median of SUVmean normalized to muscle in comparison to liver (*Z* = − 7.2, *p* < 0.001) and aorta (*Z* = − 7.2, *p* < 0.001). There was also an increased SUVmean of the lesion normalized to muscle in comparison to that value normalized to aorta (*Z* = − 7.2, *p* =  < 0.001). Figure 2 shows the lesion as well as the normalized values to the three backgrounds.

### Lesion SUV normalized to different body weight indices

Table 4 shows the computational results carried out for lesion normalization with respect to BMI, BSA, LBM, and BW. Friedman test showed a significant difference of lesions normalized to different body weight indices including body weight, body mass index, body surface area, and lean body mass (*χ*^2^ = 126.6, *p* < 0.001). Pairwise comparison using Wilcoxon rank test demonstrated a significant difference between each pair except those measures related to lean body mass including Janma and James formula (*Z* = − 1.58, *p* = 0.114). The median of SUVmax was significantly higher in this order BW > (James and Janma) > BMI > BSA with interquartile range of 19.0, 12.1, 11.4, 6.8, and 0.5, respectively.

**Table 4:** Statistical descriptions of lesion normalization with respect to BMI, BSA, LBM, and B.W

	BMI	BSA	LBMjames	LBMjanma.	B.W
*SUVmean*					
Mean	2.42 ± 1.86	0.15 ± 0.11	4.1 ± 3.0	4.0 ± 2.9	6.30 ± 4.66
Median	2.18	0.13	3.64	3.56	5.69
Max	9	0.54	13.7	12.3	20.5
Min	0.08	0.0	0.11	0.1	0.18
*SUVmax*					
Mean	4.80 ± 4.45	0.30 ± 0.27	8.15 ± 7.34	8 ± 7	12.58 ± 11.43
Median	3.6	0.24	5.94	5.78	9.91
Max	20.35	1.35	35.68	31.73	50.8
Min	0.12	0.01	0.17	0.16	0.27

Similarly, the group comparison of SUVmean measurements was also significant among the five different normalization schemes with (*χ*^2^ = 98.5, *p* < 0.001). The pairwise comparison of the five normalization was also significant for each pair except Janma and James lean body normalization was not significant (*Z* = − 1.77, *p* = 0.07). The medians, as well as the interquartile range among the five SUVmean normalization, were increasingly higher in this order BW > James and Janma > BMI > BSA. The interquartile range values were 7.35, 0.34, 0.31, 0.35, and 0.01, respectively.

### Correlation of SUV with body weight index

**Fig. 3:**
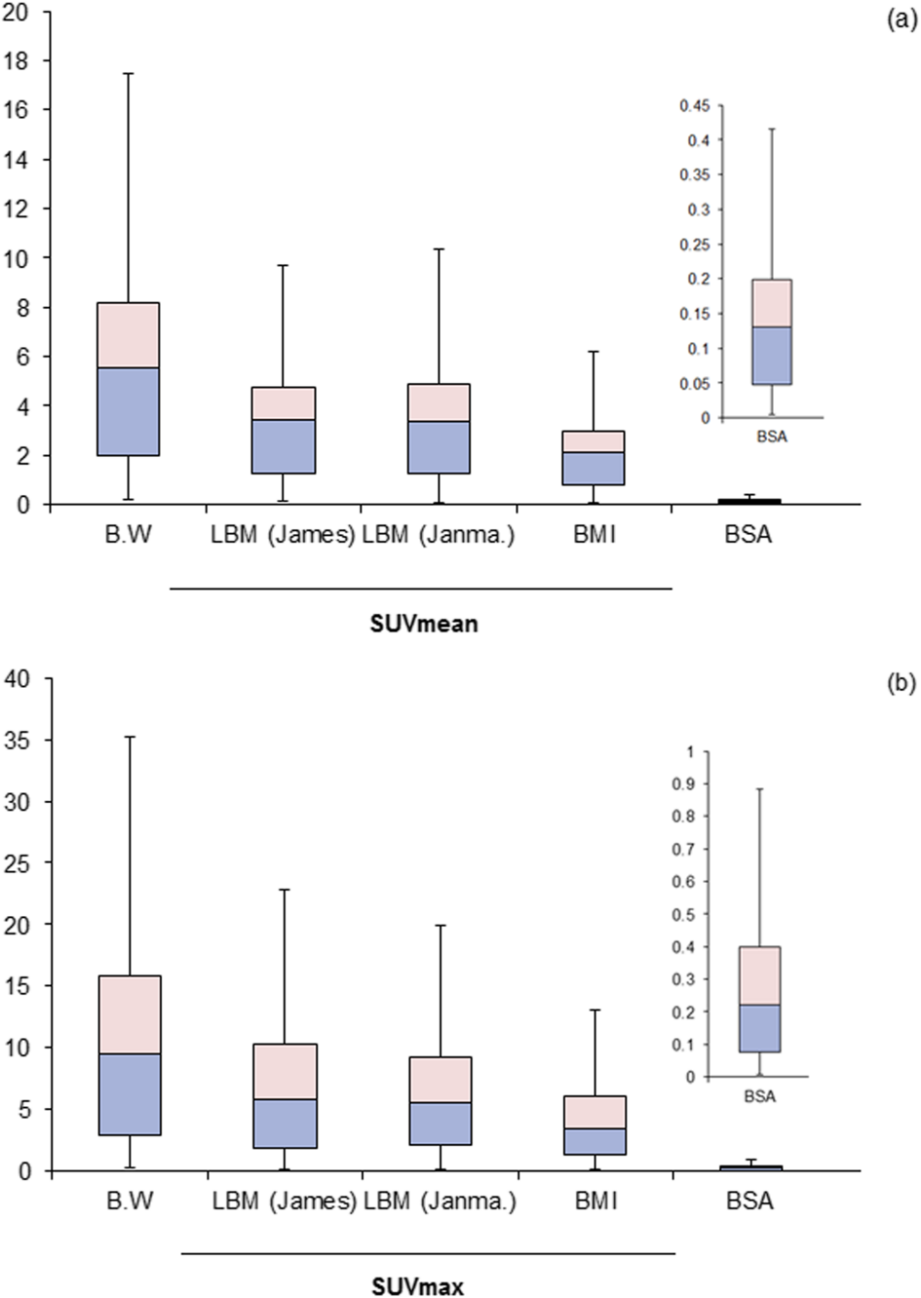
Normalization of lesion SUV to body mass index, body surface area, lean body mass, and body weight using **a** SUVmean, **b** SUVmax

Figure 3 shows the correlation relationship between the body weight index and SUV in terms of maximum and mean measurements. SUVmax normalized to body weight showed a significant correlation with body weight (*r* = 0.488, *p* = 0.003) whereas no significant correlation with James and mild correlation with Janma (*r* = 0.259, *r* = 0.347; *p* = 0.139 and *p* = 0.044, respectively).

There was also mild correlation with SUVmax normalized to BSA as well as BMI (*r* = 0.345 and *p* = 0.046) and (0.452, *p* = 0.007), respectively.

SUVmean showed a significant correlation with body weight and BMI (*r* = 0.480, *r* = 0.427 with *p* = 0.004 and *p* = 0.012) and James method (0.366, *p* = 0.033) but was not significant with BSA and Janma lean body mass (*r* = 0.057 and 0.011) with *p* = 0.012 and 0.950, respectively.

**Fig. 4:**
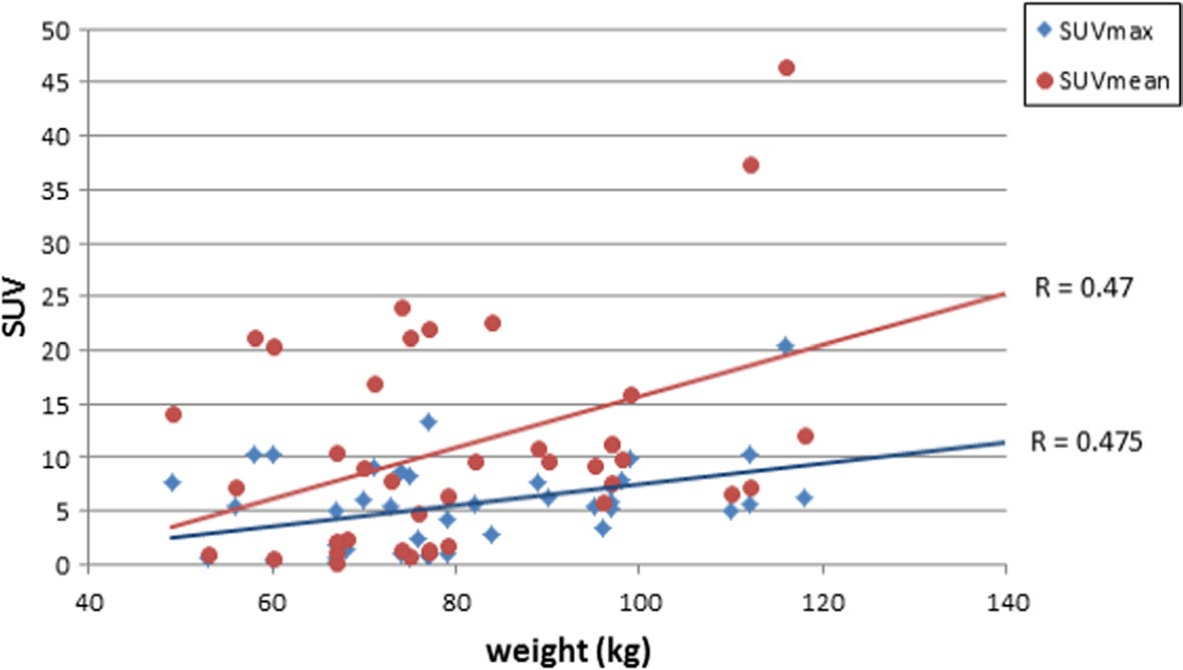
Pearson correlation coefficient between lesion SUV versus patient weight in kg with respect to B.W

**Fig. 5:**
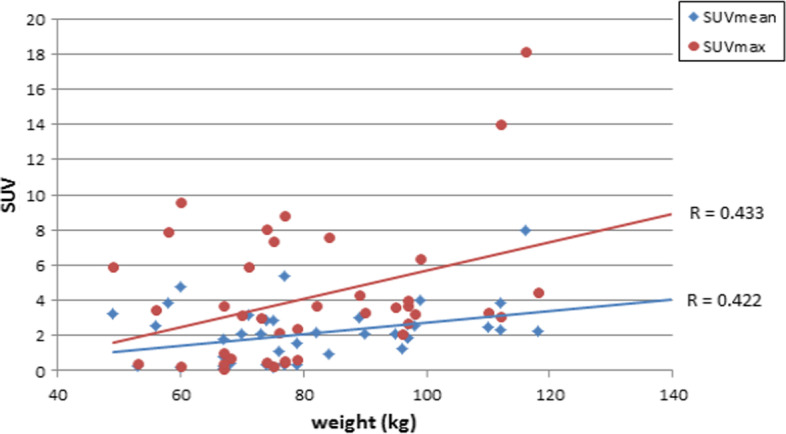
Pearson correlation coefficient between Lesion SUV versus patient's weight in kg with respect to BMI

**Fig. 6:**
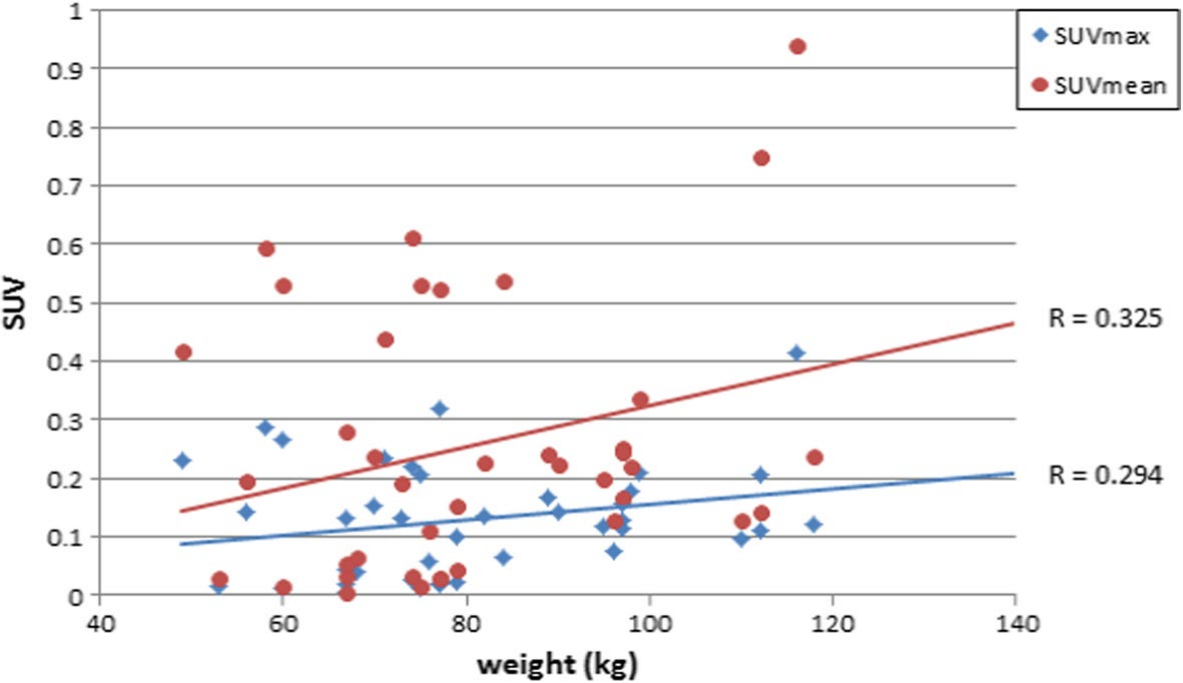
Pearson correlation coefficient between Lesion SUV versus patient's weight in kg with respect to BSA

**Fig. 7:**
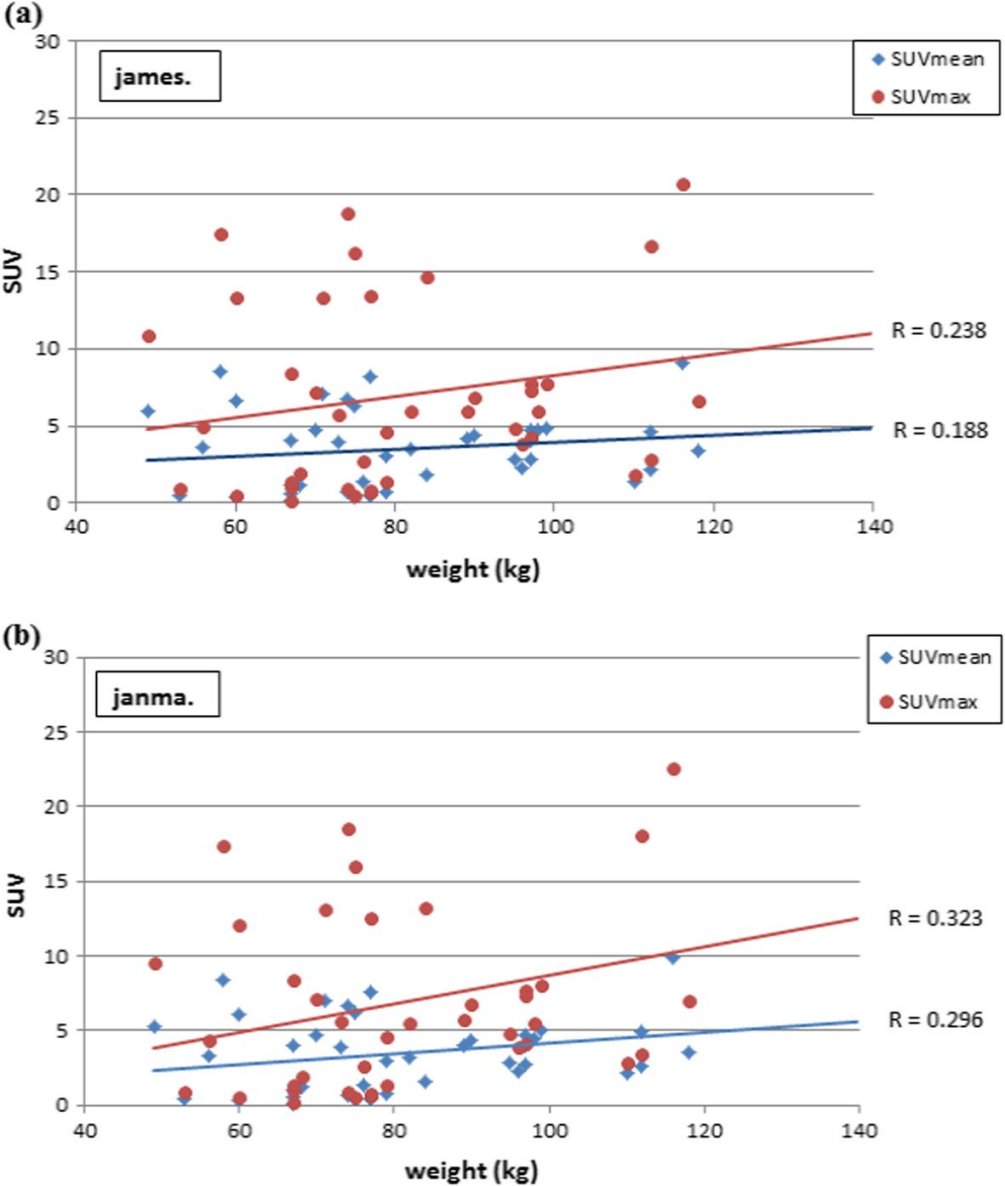
Pearson correlation coefficient between Lesion SUV versus patient’s weight in kg with respect to **a** LBMjames and **b** LBM janma, respectively

Figures 4, 5, 6, and 7 show the correlation results for each normalization method as revealed from calculations of SUVmax and SUVmean.

## Discussion

One of the most important features of PET is image quantitation that can be absolute or relative. Due to the relative complexity of absolute quantitation and determination of physiological parameters of clinical relevance, most of the routine practice is merely focused on the use of the semiquantitative SUV as surrogate measure of disease metabolic activity. PET/CT with F18-FDG has shown a great performance of detection and monitoring of patients with lymphoma (Kolthammer et al. 2014).

Although the Deauville method and visual assessment are easy to use, they are subjective and are not appropriate for clinical studies where a more objective quantitative measure is desired, except the uncommon occurrence of a complete response to therapy (Shankar et al. 2006). There are also some difficulties when the lesion uptake is close to the uptake of the reference region or when comparing the intensity of a residual uptake to that of the baseline tumor in order to classify a residual uptake within different response categories (Meignan et al. 2015). PET response is not discrete but essentially a continuum. Arbitrarily comparing the residual uptake with physiological uptake in noninvolved organs does not necessarily determine what a clinically abnormal finding is. Using a quantitative measure allows deriving optimized thresholds for normal/abnormal response based on the shape of the respective distribution (Hasenclever et al. 2014). Furthermore, comparison across a distance is challenging, especially in small residuals cases. The eye is sensitive to contrast and not to differences in intensity. So, it is recommended to read the scan using an SUV scale allowing to ‘score’ in a more objective/quantitative way the residual site.

In addition the change in SUVmax has significant advantages over the visual interpretation. It describes the kinetics of tumor activity that are could be overlooked by visual analysis, which only reflects treatment response at a specific time point (Meignan 2015). Quantitative assessment is ultimately less user-dependent and avoids optical misinterpretation caused by background activity. It can be fully automated and allows for easier comparison between centers, making multicenter trials possible (Tomasi et al. 2012). So, quantifying uptake variations is  more reproducible.

In quantitative PET/CT, the use of normal tissues as background is an important and essential quality control (Azmi et al. 2017). Several approaches have been taken to normalize oncologic lesions to normal tissues in order to stabilize and reduce test variability among several scans and/or among patient populations in multicenter trials. The use of normal liver SUVmean as background tissue has been adopted by a number of publications and guidelines including PERCIST criteria for solid tumors because it is both reliable and easy to measure and not significantly different in early versus late imaging sessions (1 h vs. 3 h imaging) (Chin et al. 2009).

However, few studies have compared different background sites. In the present study, we examined the relationship between the liver and two other sites used for background normalization namely aorta and muscle, and their impact on lesion SUV measurements. We also examined the normalization of lesion SUV and body background sites to body mass index, body surface area, lean body mass, and body weight in patients with lymphoma.

Reduced SUV variance among patients is necessary to achieve optimal determination of the metabolic activity devoid of any technical or normalization flaws. Factors affecting SUV measurements in terms of reproducibility, stability, accuracy, and consistency are discretely published (Wiyaporn et al. 2010). Among those critical factors are the selections of the lesion background region as well as body weight indices. Both appear to be important to avoid data variability and quantitative bias.

Comparison of the three different background regions revealed significant differences in their SUV measurements indicating that they are not equivalent or even interchangeable in individual patients. The liver SUV was consistently large than the aorta and muscle and both were significantly different. The liver SUV was consistent with several previous reports in the range of 2–3, on average (Meyer 2007). The variance estimate represented by the interquartile range in measurements of liver SUVmax and SUVmean was lower than that recorded for other background regions.

Additionally, normalization of SUVmax was affected by the background region such that there was a significant difference among lesions normalized to the three selected sites. In general, lesion SUVmean showed the lowest variance in comparison to lesion SUVmax probably due to the sensitive nature of SUVmax to statistics of the acquired data. In these circumstances, SUVmean could be more stable in comparison to SUVmax when a robust comparison is to be made between two different patient conditions or interscan evaluation and/or variation. More specifically the lesion SUVmean normalized to liver had the lowest median and variation among other counterparts including aorta and muscle.

Similarly, the SUVmax normalized to liver lesions was the lowest median and variation among other counterparts. The lesions/liver ratio can be viewed as a correction for normal physiological uptake elements within tumor mass or alternatively seen as how many folds malignant tissue consumes glucose in comparison to liver tissues. The stability of the liver SUV over the time curse of the study could support the use of liver as reference tissues for that purpose (Wilson 2011).

The SUVmax and SUVmean normalized to different body weight indices showed the lowest variation with BSA and BMI while being increasingly higher with lean body mass using the two methods and then highest with body weight. The median absolute median (MAM) is a measure equivalent to the coefficient of variation in nonparametric analysis and the order of normalized lesion SUVmax was analogous to the interquartile range measuring 7.73, 4.31, 3.85, 2.93, 0.19 for BW, Janma, James, BMI, and BSA, respectively. For measurements of SUVmean, the MAM values were 3.64, 0.180, 0.175, 0.15, 0.01 for BW, BMI, James, Janma, and BSA.

Correlation of the SUVmax and SUVmean with different measures of body weight indices varied between no correlation to poor and moderate significant correlation being higher with body weight and lower with lean body mass. Particularly, the SUVmax normalized to James and SUVmean normalized to lean (i.e., Janma) and BSA showed a significant independence with body weight. This would reduce systematic errors associated with elevated SUV values in patients with increased body weights.

The approach taken in this study was not to use the background site as a guide or threshold cutoff value to segment the lesion; however, the three background regions were used to normalize the lesion such that the lesion is expressed in terms of how many folds the lesion is in terms of the physiologic background activity. This method could get rid of any variations that may interfere during interscan evaluation except those related to disease modification since the background level could also be similarly affected by treatment intervention or other pathophysiological changes. The segmentation method employed in the present study was recently optimized by our group using the same scanner and imaging protocol via extensive phantom scans and 3D formulations (Abdel Gawad et al. 2019). It is characterized by considering lesion size as provided from the CT scans and the background as taken from lesion surroundings. This approach was found to provide better results in comparison to the 41% threshold since the latter was found to vary based on the lesion size and background level (Abdel Gawad et al. 2019).

Taken all together, the SUV when normalized to liver it shows less variation among patients and this was true for SUVmax with less variation appeared in SUVmean measurements. Moreover, the variation is also less when SUVmean normalization is carried out with respect to BSA and Janma lean mass and also when SUVmax is normalized to James lean body mass. This highlights future studies on the utility of using which variant of SUV with which body normalization is most appropriate not only in lymphoma but also with other oncologic PET/CT examinations.

Reduction of SUV variation would assist in improving the sensitivity of quantitation to biological changes that may happen to malignant lesions. This is of particular interest in multicenter clinical trials as results analysis should be free from different confounders reaching accurate conclusions. Future studies will be immediately conducted to utilize the current findings and account for more physical and biological confounders and in different malignancies.

## Conclusions

Comparison of the three different background regions revealed significant differences in their SUV measurements indicating that they are not equivalent or even interchangeable in individual patients. The liver SUV was consistently large than the aorta and muscle and both were significantly different. The variance estimate represented by the interquartile range in measurements of liver SUVmax and SUVmean was lower than that recorded for other background regions. The SUVmax normalized to lean (i.e., James), SUVmean normalized to lean (i.e., Janma) and BSA showed a significant independence with body weight. This would reduce systematic errors associated with elevated SUV values in patients with increased body weights.

## Data Availability

The data will be available upon reasonable request from the corresponding author.
